# Prevalence and Risk Factors of Fluid Overload in Southern Chinese Continuous Ambulatory Peritoneal Dialysis Patients

**DOI:** 10.1371/journal.pone.0053294

**Published:** 2013-01-14

**Authors:** Qunying Guo, Chunyan Yi, Jianying Li, Xiaofeng Wu, Xiao Yang, Xueqing Yu

**Affiliations:** Department of Nephrology, The First Affiliated Hospital, Sun Yat-sen University, Guangzhou, Guangdong, China; University of Louisville, United States of America

## Abstract

**Background:**

Fluid overload is frequently present in CAPD patients and one of important predictors of mortality. The aim of this study is to investigate the prevalence and associated risk factors in a cohort study of Southern Chinese CAPD patients.

**Methods:**

The patients (receiving CAPD 3 months and more) in our center were investigated from January 1, 2008 to December 31, 2009. Multi-frequency bioelectrical impedance analysis was used to assess the patient’s body composition and fluid status.

**Results:**

A total of 307 CAPD patients (43% male, mean age 47.8±15.3 years) were enrolled, with a median duration of PD 14.6 (5.9–30.9) months. Fluid overload (defined by Extracellular water/Total body water (ECW/TBW)≥0.40) was present in 205 (66.8%) patients. Univariate analysis indicated that ECW/TBW were inversely associated with body mass index (r = −0.11, *P* = 0.047), subjective global assessment score (r = −0.11, *P* = 0.004), body fat mass (r = −0.15, *P* = 0.05), serum albumin (r = −0.32, *P*<0.001), creatinine (r = −0.14, *P* = 0.02), potassium (r = −0.15, *P* = 0.02), and residual urine output (r = −0.14, *P* = 0.01), positively associated with age (r = 0.27, *P*<0.001), Chalrlson Comorbidity Index score (r = 0.29, *P*<0.001), and systolic blood pressure (r = 0.22, *P*<0.001). Multivariate linear regression showed that lower serum albumin (β = −0.223, *P*<0.001), lower body fat mass (β = −0.166, *P* = 0.033), old age (β = 0.268, *P*<0.001), higher systolic blood pressure (β = 0.16, *P* = 0.006), less residual urine output (β = −0.116, *P* = 0.042), and lower serum potassium (β = −0.126, *P* = 0.03) were independently associated with higher ECW/TBW. After 1 year of follow-up, the cardiac event rate was significantly higher in the patients with fluid overload (17.1% vs 6.9%, *P* = 0.023) than that of the normal hydrated patients.

**Conclusions:**

The prevalence of fluid overload was high in CAPD patients. Fluid overload in CAPD patients were independently associated with protein-energy wasting, old age, and decreased residual urine output. Furthermore, CAPD patients with fluid overload had higher cardiac event rate than that of normal hydrated patents.

## Introduction

Fluid overload is a common complication of peritoneal dialysis [1], which is closely related to hypertension [2], [3], cardiac dysfunction [4], [5], inflammation [6], and mortality in dialysis patients [7], [8]. In PD patients, measures of improving fluid status include attempt by dialysis prescription to optimize sodium and water removal, emphasis limits on sodium and water intake, and administration diuretics to enhance residual urine output. However, the crucial barrier to improve fluid management is the limitation of indentifying early or occult overhydration, but not the lack of strategies to enhance fluid removal [9].

Usually physicians estimate hydration status by clinical parameters, such as extremities edema, weight gain or increased blood pressure [10], which provide a useful, but imprecise picture of fluid status. Multi-frequency bioelectrical impedance analysis (BIA) is the most extensively studied technique to assess hydration in patients. It involves measurements at a large number of frequencies, with mathematical modeling of the data to estimate ECW (theoretic resistance at a frequency of zero) and TBW (theoretic resistance at infinite frequency), and ECW being the marker of hydration [11]. Recent studies demonstrate it is of potential value to provide precise and sensitive method for detecting longitudinal changes in hydration [9], [12], [13].

Protein-energy wasting is a major negative prognostic factor in dialysis patients, which may worsen patient outcome by aggravating existing inflammation, accelerating atherosclerosis and increasing susceptibility to infection [14]. Although it was reported that serum albumin, as a nutritional marker, correlated well with multi frequency BIA parameters [15], whether protein-energy wasting is the main contributor to fluid overload in PD patients remains unclear. The present study attempted to assess the prevalence and correlation factors of fluid overload in the southern Chinese CAPD patients and investigate the relationship between fluid overload and malnutrition.

## Methods

### Objectives

This was a cross-sectional, observational, single center study. We hypothesized that protein-energy wasting was a main contributor to fluid overload in PD patients. Thus the primary aim of this study was to measure the hydration status in prevalent CAPD patients and investigate the association of fluid status with protein-energy wasting, also patient demographics, biochemical markers, PD prescription, and medications.

### Participants

The patients inclusion criteria were as follows: (1) undergoing CAPD ≥3 month; (2) age >18 years; (3) signed informed consent form. The exclusion criteria were as follows: (1) patients with pacemakers; (2) patients with amputation; (3) patients who were not able to accomplish the analysis of body composition in standing position for 3 minutes. Totally 585 patients were followed up regularly at the clinic from Jan 1 2008 to Dec 31, 2009. 160 patients refused to participate in this study. 118 patients were excluded from this study according to the exclusion criteria. Finally 307 PD patients were recruited.

### Ethics

The study protocols were approved by the Ethics Committee of The First Affiliated Hospital of Sun Yat-sen University. Informed consent was obtained from each patient.

### Investigations Undertaken

Patients were evaluated during a routine clinical visit, from January 1, 2008 to December 31, 2009.

Patients provided a 24-hour collection of urine and effluent, completed on the morning of the study visit. Then the fluid was analyzed for volume, urea, and creatinine content; Kt/V and residual renal function (RRF) were calculated using PD Adequest software (Baxter Healthcare, USA). D/Pcr was determined based on results of the last available PET test preceding the BIA measurement. Ultrafiltration and residual urine output were calculated from the patient’s charts as a daily mean of ultrafiltration and urine obtained during the last week preceding the measurement. Solution glucose concentration (%) was calculated by dividing the daily total amount of glucose in dialysis solution by solution volume. At the same time, the data of patient demographic information, PD prescription, medications were collected.

Sitting blood pressure was measured using equipment that meets certification criteria, and recorded as the mean of two consecutive measurements with at least 2 minutes interval.

Blood was taken from patients to measure the following biochemical markers: hsCRP, hemoglobin, creatinine, albumin, transferrin, total cholesterol, triglyceride, serum phosphate, serum calcium, intact-PTH, serum sodium, serum chloride, serum potassium, carbon dioxide, serum glucose.

All the patients were regularly followed up and educated to restrict salt intake of approximate 5 to 6 gram per day. Patient-outcome, cardiac event rate, and residual renal function change after 1 year of follow-up were also recorded. Cardiac event was defined as myocardial infarction, angina, and congestive heart failure.

### Bioimpedance Analysis

TBW, ECW, intracellular water (ICW), and body composition was measured by multi-frequency bioelectrical impedance model InBody 720 (Biospace, Seoul, Korea). InBody 720 uses state –of –the-art technology, and 8-point tactile electrode system that measures the total and segmental impedance and phase angle of alternating electric current at six different frequencies (1 kHz, 5 kHz, 50 kHz, 250 kHz, 500 kHz, and 1000 kHz). Peritoneal dialysis fluid was not drained from the abdomen as it has been previously shown no significant effect on BIA measures [2], [16], [17]. Impedance measurements were made with the subject standing in an upright position, on foot electrodes in the platform of the instrument. The subject stood on the four foot electrodes: two oval shape electrodes and two heel shape electrodes, and gripped the two Palm-and Thumb electrodes in order to yield two thumb electrodes and two palm electrodes, without shoes or excess clothing. The skin and the electrodes were pre-cleaned using the specific electrolyte tissue according to the manufacturer’s instructions. Prior to this, height was recorded to the nearest 0.1 cm. All the subjects were instructed to fast and to avoid exercise 8 h before measurement and had been resting for at least 30 min before measurement. All the body composition data were performed in the instrument by inner software and typed in the result sheet immediately after measuring. The software provides a plot of reactive and resistive components of the measured impedance at each frequency, as well as body weight, fat-free mass (FFM), TBW, ICW, ECW, segmental fluid distribution, fat mass (FM), body cell mass (BCM) and body mass index (BMI). This tool has been assessed in normal populations, renal transplant patients, hemodialysis patients, and PD patients, and closely correlates with the gold standard measurement by isotope dilution [18], [19], [20], [21], [22].

In this study, the ratio of ECW/TBW ≥0.4 was used to define overhydration, which was based on a fluid status measurement in 6520 normal healthy Koreans. The threshold of 0.4 represented mean +2SD, i.e. >95th percentile for Asian normal people.

### Measurement of Nutrition Status

Subjective Global Assessment (SGA) was performed by one experienced dialysis nurse blinded to all clinical and biochemical variables of the patients. The method is based on patient’s history and physical examination as described by Detsky [23]. The history focuses on gastrointestinal symptoms (anorexia, nausea, vomiting, and diarrhea) and weight loss in the preceding 6 months. The physical examination is graded by muscle wasting, loss of subcutaneous fat and the presence of ankle edema. The nutritional status was scored on the 7-point scale of the SGA. SGA scores of 5 or lower were defined as malnutrition.

### Measurement of Comorbidity

The comorbidity score of each patient was determined according to the Chalrlson Comorbidity Index (CCI). The CCI is one of the most commonly used comorbidity models which based on comorbid conditions with varying assigned weights, resulting in a composite score. The CCI was calculated by assigning a weight of 2 to diabetes, stroke, renal insufficiency, and malignancy, and a weight of 1 to the other comorbidities [24], [25]. Cardiovascular disease (CVD) was defined as myocardial infarction, angina, or history of congestive heart failure, cerebrovascular event, and peripheral vascular disease. Of all the patients, 10 patients had myocardial infarctions, 71 patients suffered from congestive heart-failure, 56 patients had clinical signs of peripheral atherothrombotic vascular disease and 23 patients suffered from cerebrovascular disease. Seven patients had been diagnosed with chronic lung disease, 3 had moderate or severe liver illness, 1 had gastrointestinal ulcer, and 4 patients suffered from malignant tumor.

### Statistical Analyses

The patient’s characteristics were presented as mean ± SD for continuous variables, and percentages and frequencies for categorical variables. The independent sample t-test was used for normally distributed continuous variables between overhydrated (ECW/TBW≥0.4) and normal hydrated (ECW/TBW<0.4) patients. A comparison of non-normally distributed continuous variables was performed using Mann-Whitney *U*-test. For categorical variables, the Chi-square test was used. The bivariate correlation test was performed to examine the associations of demographic and biochemical data with ECW, ECW/height, and ECW/TBW. Spearman’s correlation coefficients were used for non-normally distributed variables and Pearson’s correlation coefficients were used for normally distributed variables. Factors that reached statistical significance were selected for further multivariable analyses. As the ECW/TBW data was normally distributed in this study, multiple linear regressions were performed for multivariate analysis to explore the significant risk factors of ECW/TBW. All calculation was performed with SPSS 13.0. *P* value of less than 0.05 was considered to be significant.

## Results

### Prevalence of Fluid Overload (Defined by ECW/TBW≥0.40) and Edema (by Physical Examination)

A total of 307 CAPD patients (43% male ) were enrolled, their mean age was 47.8±15.3 years old, with a median PD duration of 14.6 (5.9-30.9) months. Clinical, demographic and laboratory characteristics of the 307 CAPD patients were shown in Table 1. Fluid overload was present in 205 (66.8%) CAPD patients, while edema (which was assessed by physical examination) was present in 138 (138/307, 45%) CAPD patients (*P*<0.001). Of note, 88 (88/169, 52%) patients without edema was diagnosed as fluid overload by BIA. While in the 138 CAPD patients who was clinically diagnosed as edema, 26 (26/138, 19%) patients were not fluid overload according to the BIA measurement (data not shown).

**Table 1:** Clinical, demographic and laboratory characteristics in CAPD patients with fluid overload and normal status.

	All patients 3	ECW/TBW ≥0.4	ECW/TBW<0.4	*P* value
	N = 307	N = 205	N = 102	
**Baseline demographics**				
Male (% )	132/307 (43% )3	82/205 (40% )	50/102 (49% )	P = 0.133
Age (years )	47.8±15.3	50.4±15.7	42.7±13.2	P<0.001**
Duration of CAPD (months )	14.6 (5.9 -30.9 )	14.0 (5.9-30.4)	15.1 (5.4-32.6)	P = 0.538
BMI (kg/m^2^)	22.7±3.9	22.9±3.8	22.6±3.9	P = 0.497
Diabetes (%)	49/307 (16%)	39/205 (19%)	10/102 (9.8%)	P = 0.039*
Systolic blood pressure (mmHg)	137±22	140±22	132±22	P = 0.007**
Residual urine volume (ml/24 hours)	522±459	500±450	565±477	P = 0.254
D/Pcr	0.68±0.11	0.69±0.11	0.67±0.11	P = 0.96
RRF (ml/min·1.73 m^2^)	1.66 (0.31-3.28)	1.57 (0.42-3.25 )	1.77 (0.23-3.54 )	P = 0.977
SGA score≤5 percentage (%)	120/307 (39%)	90/205 (44%)	30/102 (29%)	P = 0.018*
CVD (%)	232/307 (76%)	166/205 (81%)	66/102 (65%)	P = 0.003**
CCI score	4 (3 - 6)	4 (3 - 6)	3 (3 - 5)	P = 0.02*
ECW (L)	13.9±3.1	14.1±3.2	13.4±2.7	P = 0.09
ICW (L)	20.8±4.5	20.5±4.6	21.3±4.3	P = 0.932
ECW/Height	0.085±0.17	0.087±0.018	0.082±0.014	P = 0.032*
ECW/TBW	0.40±0.13	0.41±0.01	0.39±0.01	P<0.0001**
**PD prescription**				
Solution glucose concentration (%)	1.5 (1.5-1.8)	1.5 (1.5-1.83)	1.5 (1.5-1.75)	P = 0.05*
KT/V	2.1 (1.8-2.6)	2.2 (1.8-2.6)	2.1 (1.7-2.5 )	P = 0.567
Ultrafiltration (ml/24 hours)	496.1±475.58	483±468	520±493	P = 0.531
Salt restriction (yes/no)	177/130	116/89	61/41	P = 0.945
**Biochemical markers**				
HsCRP (mg/L)	2.0 (0.8-8.3)	2.23 (0.97-9.36)	1.71 (0.64-5.07)	P = 0.08
Creatinine (umol/L)	923±353	868±336	1032±362	P = 0.001**
Albumin (g/L)	39 (36-42)	38 (35-41)	41 (38-43)	P<0.001**
Transferrin (g/L)	2.3 (1.9-2.6)	2.2 (1.9-2.6)	2.4 (2.1-2.6)	P = 0.016*
Phosphate (mmol/L)	1.67±0.58	1.61±0.54	1.77±0.63	P = 0.03*
Potassium (mmol/L)	3.8 (3.3-4.2)	3.7 (3.3-4.1)	4.0 (3.4-4.4)	P = 0.02*
Carbon dioxide (mmol/L)	25±4	26±4	25±3	P = 0.01**
Glucose (mmol/L)	4.5 (3.9-5.3)	4.6 (4.1-5.7)	4.3 (3.8-4.8)	P = 0.006**
**Medications**				
Number of anti-hypertension drugs	2 (1-3)	2 (1-3)	2 (1-3)	P = 0.517
calcium channel blockers	199/307 (65%)	144/205 (70%)	55/102 (54%)	P = 0.26
Diuresis (%)	19/307 (6%)	12/205 (6%)	7/102 (7%)	P = 0.591
Dose of diuretics (mg/day)	80 (40, 100)	80 (40, 120)	40 (20, 80)	P = 0.062

Notes: *P<0.05, **P<0.01. SGA = subjective global assessment; CVD = cardiovascular disease; CCI = Charlson Comorbidity Index; ECW = extracellular water; ICW = intracellular water; TBW = total body water; hsCRP = high-sensitivity C-reactive protein; BMI = body mass index; RRF = Residual renal function.

Of the 278 non-studied patients, 66% were male, 18% diabetic patients. Their mean age was 53. 2±16.0 years old, with a median PD duration of 1.73 (1.37-7.20) months. Compared with the non-studied patients, the studied patients were younger, had obvious lower male patients proportion, longer PD duration, and lower residual urine volume (data not shown). While the proportion of diabetic patients, and the proportion of patients with edema (46% vs 44%) by physical examination was comparable in the studied and non-studied patients.

### Receiver – Operating Characteristic Curve (ROC) Analysis of Edema

**Figure 1:**
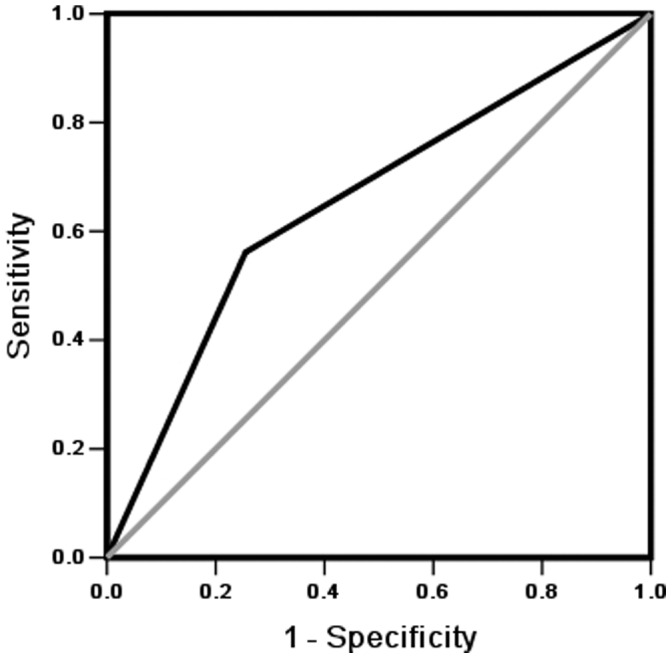
ROC analysis of edema for fluid overload (AUC = 0.653, sensitivity 0.562, specificity 0.745, P<0.001).

As shown in Figure 1, we used ROC analysis to calculate the sensitivity and specificity of edema (by physical examination) as a diagnostic tool to diagnose fluid overload (defined by ECW/TBW ≥0.40) in 307 CAPD patients (area under the concentration curve, AUC = 0.653, sensitivity 0.562, specificity 0.745, P<0.001).

### Characteristics of CAPD Patients with Fluid Overload

The clinical, demographic and laboratory characteristics were compared between the CAPD patients with fluid overload and patients without overhydration as shown in Table 1. Compared with normal hydrated patients, patients with fluid overload were older (50.4±15.7 vs 42.7±13.2 years, *P<*0.001), had higher diabetic percentage (19% vs 9.8%, *P = 0.039*), higher malnourished percentage (SGA score ≤5) (44% vs 29%, *P = 0.018*), higher CVD percentage (81% vs 65%, *P = 0.003*), higher CCI score (4 vs 3, *P = 0.02*), and higher systolic blood pressure (140±22 vs 132±22 mmHg, *P = *0.007), but had lower serum albumin level (38 (35–41) vs 41 (38–43) g/L, *P*<0.001), lower serum potassium (3.7 (3.3–4.1) vs 4.0 (3.4–4.4) mmol/L, *P = *0.018 ), lower serum creatinine (868±336 vs 1032±362 µmol/L, *P = *0.001). There was no significant difference in the proportion of calcium channel blockers using (70% vs 54%, P = 0.26) and diuretics using (6% vs 7%, P = 0.59) in both two groups of patients. All the patients in this study used only one kind of loop diuretics (furosemide), and the dosage of furosemide was not significantly different between the two groups (80 (40, 120) vs 40 (20, 80), P = 0.062) (as shown in Table 1).

### Fluid Status in Different Subgroups of Patients

**Figure 2:**
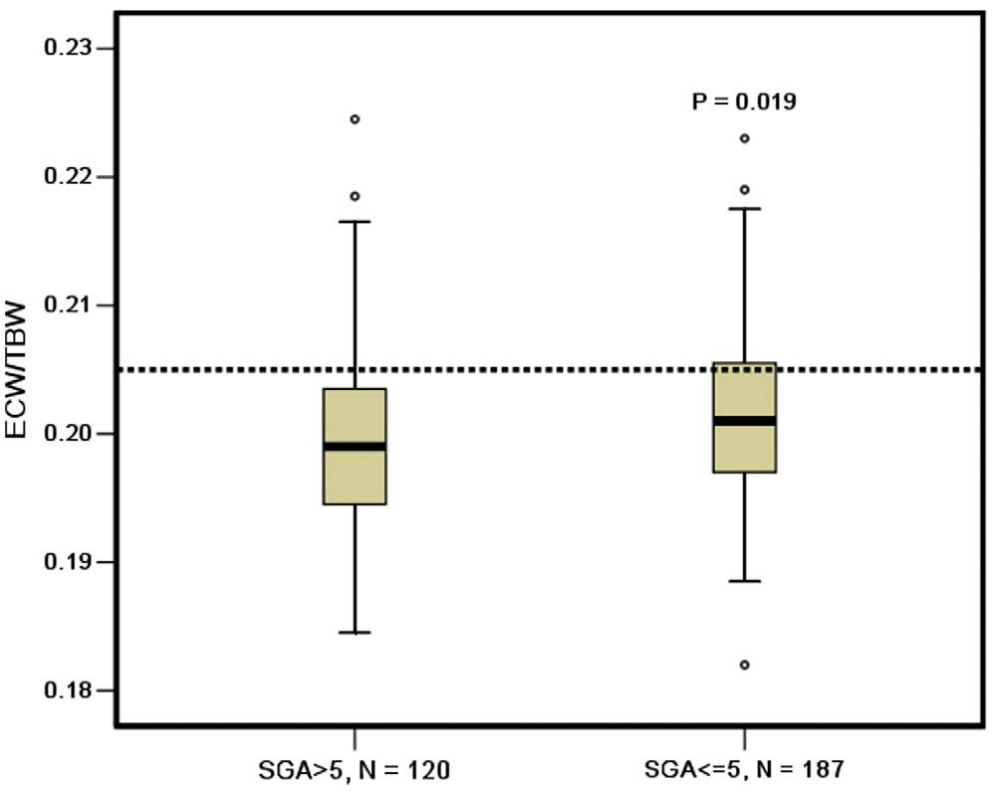
ECW/TBW in the CAPD patients with and without malnutrition. *, P<0.05; SGA, subjective global assessment.

**Figure 3:**
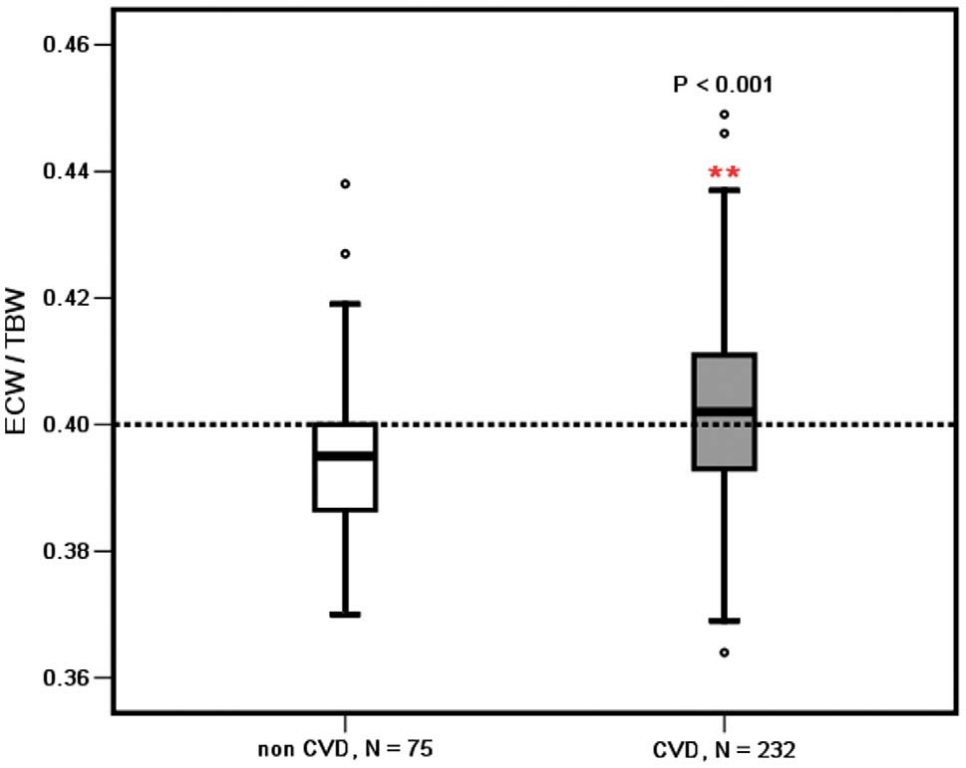
ECW/TBW in the CAPD patients with and without CVD. **, P<0.01; CVD, cardiovascular disease.

**Figure 4:**
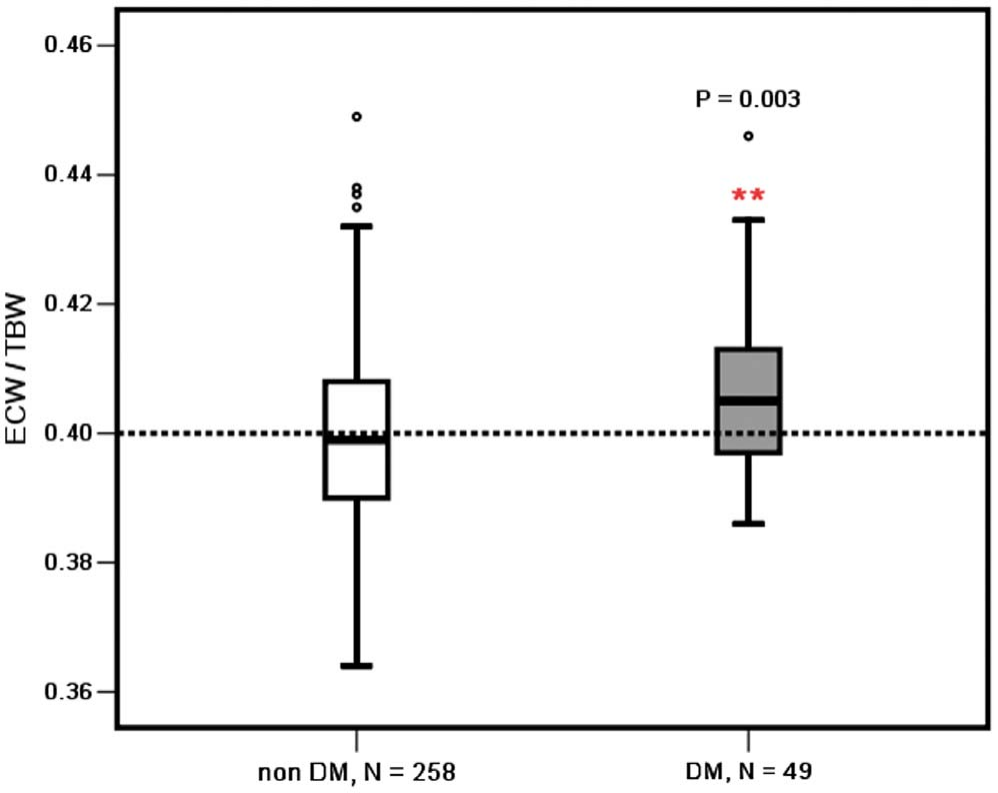
ECW/TBW in the diabetic and non-diabetic CAPD patients. **, P<0.01; DM: diabetes mellitus.

The ECW/TBW ratio of malnourished patients, CVD patients, and diabetic patients was significantly higher than that of the patients without malnutrition (0.403±0.013 vs 0.399±0.013, *P = *0.019), non CVD patients (0.402±0.013 vs 0.396±0.011, *P<*0.001), and non diabetic patients (0.406±0.012 vs 0.399±0.013, *P = *0.003), respectively, as shown in Figures 2, 3, and 4.

### Univariate Correlations for ECW/TBW in CAPD Patients

**Figure 5:**
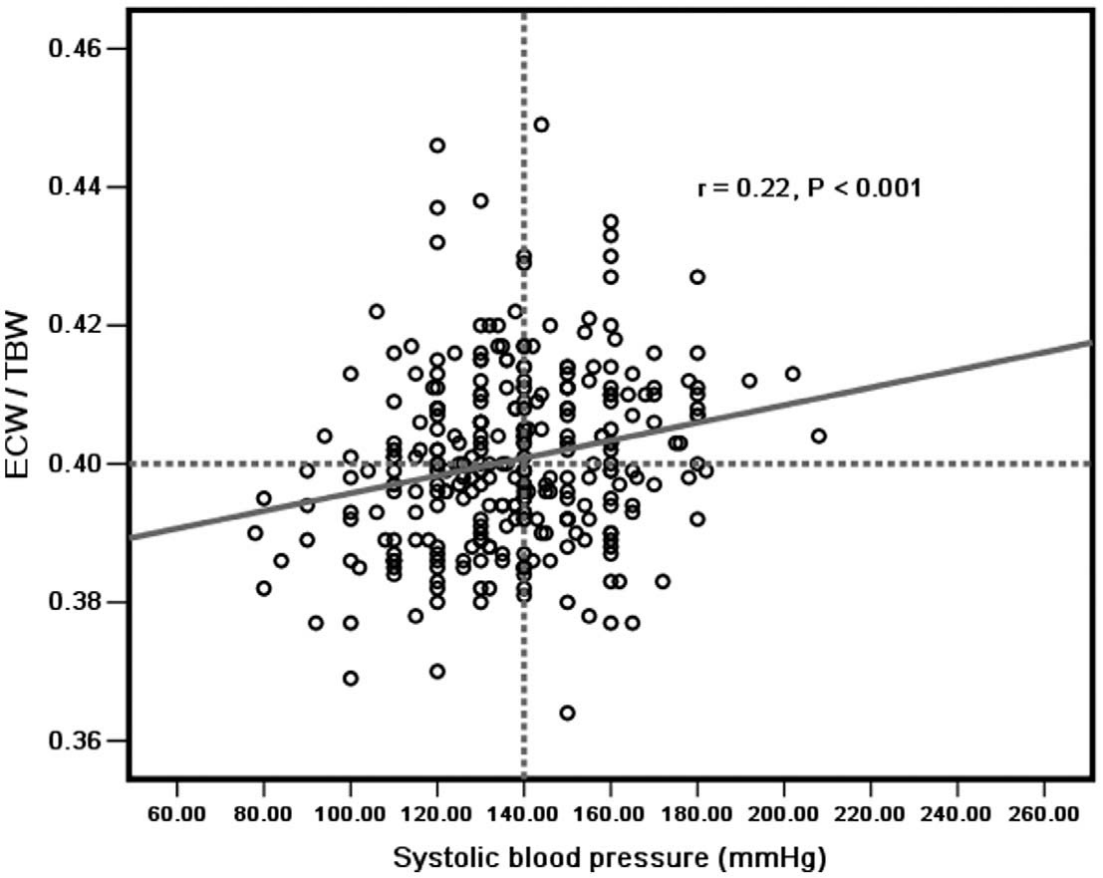
ECW/TBW was positively correlated with systolic blood pressure (mmHg).

**Figure 6:**
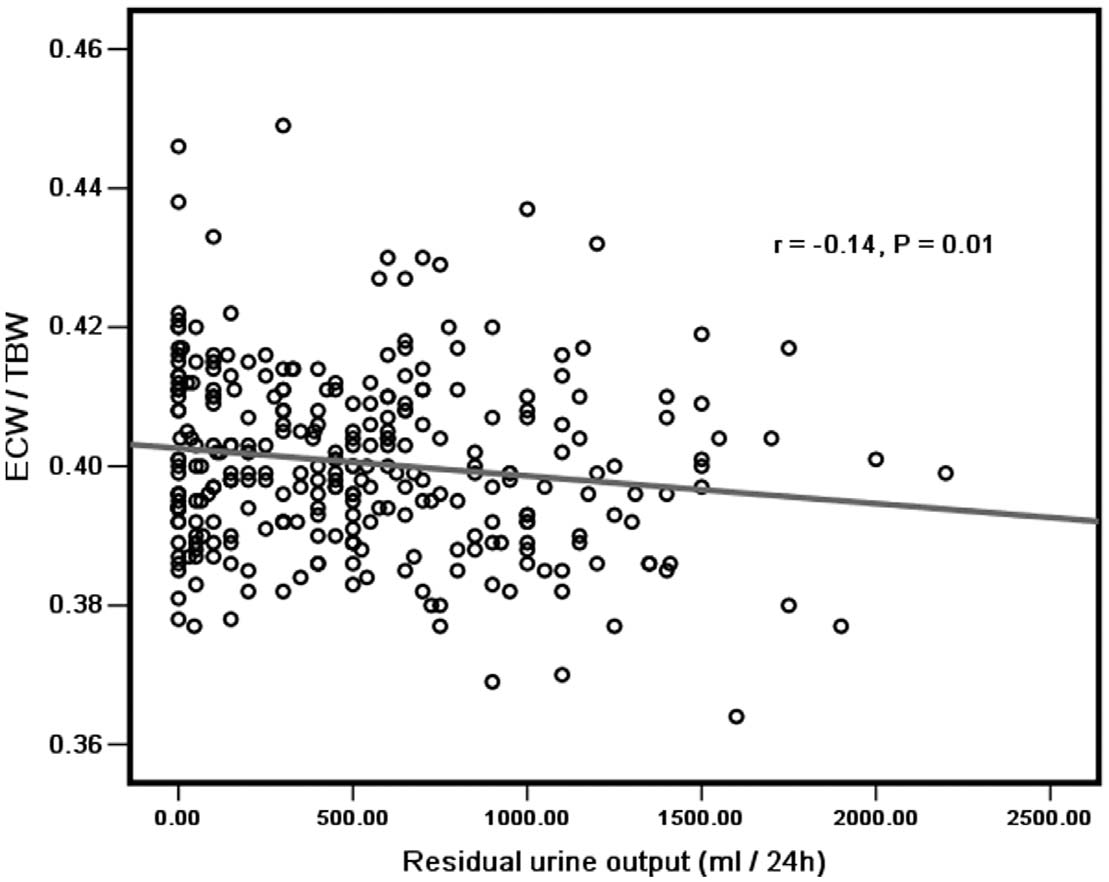
ECW/TBW was negatively correlated with residual urine output (ml/24 **h).**

Univariate correlation analysis indicated that ECW/TBW were inversely associated with BMI (r = −0.11, *P = *0.047), SGA score (r = −0.17, *P = *0.004), body fat mass (r = −0.15, *P = *0.05), serum albumin (r = −0.32, *P*<0.001), serum creatinine (r = −0.14, *P = *0.02), serum potassium (r = −0.15, *P = *0.02), and residual urine output (r = −0.14, *P = *0.01), but positively associated with age (r = 0.27, *P*<0.001), CCI score (r = 0.29, *P*<0.001), solution glucose concentration (r = 0.20, *P*<0.001), and systolic blood pressure (r = 0.22, *P*<0.001), but not hs-CRP, ultrafiltration, KT/V, D/Pcr, diuretic or anti-hypertension drugs using, as shown in Table 2, Figures 5 and 6.

**Table 2:** Variables correlation with ECW, ECW/height, ECW/TBW.

	ECW	ECW/height	ECW/TBW
Age (years)	−0.03	0.02	0.27**
BMI (kg/m^2^)	0.52**	0.69**	−0.11*
Duration of CAPD (months)	0.22**	0.21**	0.03
Systolic blood pressure (mmHg)	0.21**	0.21**	0.22**
Ultrafiltration (ml/24 hours)	0.13*	0.12	0.04
Residual urine volume (ml/24 hours)	−0.11	−0.11	−0.14*
D/Pcr	0.10	0.10	0.13
RRF (ml/min·1.73 m2)	−0.17**	−0.15*	−0.11
KT/V	−0.30**	−0.24**	−0.06
SGA score	0.08	0.07	−0.17**
CCI score	0.03	0.08	0.29**
Edema^a^	0.15*	0.17**	0.39**
Body fat mass (kg)	−0.11	−0.07	−0.15*
Solution glucoseconcentration (%)	0.16**	0.13*	0.20**
hsCRP (mg/L)	0.06	0.06	0.13
Albumin (g/L)	−0.09	−0.12*	−0.32**
Creatinine (µmol/L)	0.31**	0.25**	−0.14*
Potassium (mmol/L)	0.16**	0.14*	−0.15*
Carbon dioxide (mmol/L)	−0.00	0.01	0.15*
Glucose (mmol/L)	0.01	0.02	0.25**

Notes: *P<0.05, **P<0.01. SGA = subjective global assessment; CVD = cardiovascular disease; CCI = Charlson Comorbidity Index; ECW = extracellular water; TBW = total body water; hsCRP = high-sensitivity C-reactive protein; BMI = body mass index; RRF = Residual renal function; a, Edema was assessed by physical examination.

### Multivariate Linear Regression Analysis for Predictors of ECW/TBW in CAPD Patients

In multivariate linear regression, only old age (β = 0.268, *P*<0.001), lower serum albumin (β = −0.223, *P*<0.001), lower body fat mass (β = −0.166, *P = *0.033), higher systolic blood pressure (β = 0.16, *P = *0.006), less residual urine output (β = −0.116, *P = *0.042), and lower serum potassium (β = −0.126, *P = *0.03) were independently associated with higher ECW/TBW (as shown in Table 3).

**Table 3:** Predictors of ECW/TBW in CAPD patients by multivariate linear regression analysis.

	Unstandardized Coefficients (B)	Standardized Coefficients (Beta)	*P* value	95% Confidence Interval for B
Age (years)	0.000	0.268	<0.001**	0.000–0.000
Sex	0.002	0.058	0.309	−0.001–0.005
BMI	0.000	−0.085	0.253	−0.001–0.000
Diabetes	0.003	0.073	0.254	−0.002–0.007
CCI	0.000	0.066	0.363	−0.001–0.001
Systolic blood pressure (mmHg)	9.80E-005	0.160	0.006**	0.000–0.000
Residual urine volume (mL)	−3.4E-006	−0.116	0.042*	0.000–0.000
Albumin (g/L)	−0.001	−0.223	<0.001**	−0.001–0.000
Body fat mass (kg)	0.000	−0.166	0.033*	−0.001–0.000
Potassium (mmol/L)	−0.002	−0.126	0.03*	−0.001–0.000

Notes: *P<0.05, **P<0.01. CCI = Charlson Comorbidity Index; ECW = extracellular water; TBW = total body water; BMI = body mass index.

### Clinical Outcome after One Year of Follow-up

After one year of follow-up, 16 (5.2%) patients died, 8 (2.6%) patients transferred to hemodialysis, 22 (7.2%) patients received kidney transplantation, 1 (0.3%) patient lost of follow-up. As shown in the Table 4, the 1- year patient survival rate in the normal hydrated patients is higher than that of the overhydrated patients (98.0% vs 93.2%, Log Rank = 0.086), but not significantly. Also, there was no significant difference of 1- year technique survival rate between the two groups of patients (90.2% and 95.1%, Log Rank = 0.179). Nevertheless the cardiac event rate was significantly higher (17.1% vs 6.9%, *P = *0.023) in the patients with fluid overload than that of the patients with normal hydration. While no significant difference in the CVD mortality was found between the two groups of patients (2.0% vs 4.4%, Log Rank = 0.302). In addition, no difference was found in the decrease of residual renal function in 1 year in the patients with overhydration and that of the patients without overhydration (0.45 vs 0.26 ml/min·1.73 m^2^, *P = *0.603).

**Table 4:** Clinical outcome at 1 year follow-up.

	ECW/TBW ≥0.4	ECW/TBW <0.4	*P* value
	N = 205	N = 102	
1- year patient survival (%)	93.2%	98.0%	0.086
1- year technique survival (%)	90.2%	95.1%	0.179
CVD mortality	4.4%	2.0%	0.302
Cardiac event rate (%)	17.1%	6.9%	0.023*

Notes: *P<0.05; CVD = cardiovascular disease.

Seventy-nine patients had persistent overhydration, 187 patients had intermittent overhydration, while 41 patients were maintained euvolaemic over the 1 year follow-up period. There was no significant difference in the decrease of residual renal function in the patients who were persistently overhydrated and those that were maintained euvolaemic over the follow-up period (0.75 vs 0.26 ml/min·1.73 m^2^, *P = *0.089).

## Discussion

In the present study, the prevalence and risk factors of fluid status were investigated in a cohort study of CAPD patients. We found that the prevalence of fluid overload by bioimpedance analysis in CAPD patients was 66.8%. Fluid overload in CAPD patients were independently associated with protein-energy wasting, old age, higher systolic blood pressure, hypokalemia, and decreased residual urine volume, but not peritoneal transport characteristics and inflammation.

In PD patients, practitioners rely most on routine physical examination, observation of body weight, blood pressure, and fluid output to diagnose fluid overload [10], which provide a useful, but imprecise picture of fluid status. BIA is the most extensively studied and most promising technique for routine monitoring of fluid status and nutrition in dialysis patients [26], [27], [28], [29], [30]. The present study showed the prevalence of fluid overload by bioimpedance analysis in CAPD patients was 66.8% in this study, which was notablely higher than the diagnosis rate of 45% by the routine physical examination. Our results support BIA can be use to indentify some clinical occult fluid overload patients before notable clinical edema appear, which enables the appropriate therapeutic changes. Of the 138 CAPD patients with clinical edema, 26 (19%) patients were clinically diagnosed as edema, but not in fluid overload status according to the BIA measurement. Theoretically, under-evaluation of ECW/TBW may happen to athletes, who have relatively more skeletal muscle cells, and less extracellular water. Compared with the other 112 patients who were diagnosed as both edema and fluid overload by BIA, these 26 patients were younger (44.7±12.1 vs 51.7±15.5, P = 0.033), had lower proportion of DM (7.7% vs 25%, P = 0.054) and less CVD history (58.3% vs 85.8%, P = 0.002). More importantly, they also had significantly higher skeletal muscle mass (27.2±5.1 vs 24.7±5.8, P = 0.043), thus more intracellular water (22.4±3.9 vs 20.6±4.4, P = 0.06) and body protein mass (9.7±1.7 vs 8.9±1.9, P = 0.056). Thus these data may explain the reason of the underestimation of ECW/TBW in this group of patients.

We clearly demonstrated our hypothesis that a close relationship existed between protein-energy wasting and overhydration in a cohort of CAPD patients. As showed in the study, the overhydrated patients had significantly higher malnourished percentage, lower serum albumin and creatinine level than that of the normal hydrated patients. Univariate analysis showed the ECW/TBW ratio was inversely associated with BMI, SGA score, body fat mass, serum albumin, and creatinine. Further multivariate analysis confirmed that lower serum albumin and body fat mass were independently associated with overhydration. As it is well known that serum albumin, serum creatinine, BMI, body fat mass, and SGA are good surrogate markers of protein-energy wasting [31], a PD patient developing protein-energy malnutrition may gradually acquire ECW accumulation to balance loss of body cell mass or fat mass. Lower serum albumin level of malnourished patients not only caused a reduced colloid osmotic pressure and certainly aggravated the fluid retention in the tissue space, but also was a consequence of hemodilution due to fluid overload. Although worsening nutrition status may contribute to deteriorating fluid retention, whether improving nutritional status could help to improve fluid overload in dialysis patients needs further research.

Another interesting finding from this study was that decreased residual urine volume, but not ultrafiltration, RRF, or overall solute clearance (KT/V), play a possible pathogenic role in volume overload independent of diuretic usage and other confounders. Indeed, a close look at the reanalysis of the CANUSA data reveals that it was not the additional solute clearance obtained by renal function, but the urinary volume excreted that was the driving force for the relative risk of death, pointing to the fact that “volume status” is more important than small solute clearance [32], [33]. The similar finding was reported by Ates et al [34], their finding suggests that removal of sodium and fluid is a predictor of mortality in PD patients, whereas Kt/V and total creatinine clearance are not risk factors. Our finding is contradictory with the report from the European Body Composition Monitoring Study Cohort [2], which showed that neither urinary output nor ultrafiltration contributed to the fluid balance. The reason of this discrepancy is not clear. We speculate that these different findings may relate to the high percentage of polyglucose usage (63.7%) and automated PD (APD) modality (53.1%) in these European group of patients, while only CAPD modality with glucose solution was performed in our patients. Indeed, APD is associated with lower sodium removal and may cause a more rapid loss of residual renal function [35], [36], [37], both of which may lead to an increase in extracellular fluid in these patients. However, Davison SN et al. recently compared extracellular fluid volume (ECFV) in patients who received CAPD and cycler-assisted peritoneal dialysis (CCPD) with fewer night cycles and icodextrin using for long daytime, no difference in the ratio of ECFV/TBW was found in these two groups of patients [38].

Fluid overload contributes to the development of hypertension in PD patients [2], [3], [39]. We also demonstrated a direct correlation between systolic blood pressure and tissue hydration as other authors reported [2]. It is well known that systolic blood pressure is a high risk factor for CVD and CVD caused death. Our findings also showed that the CAPD patients with overhydration had higher CVD percentage than that in patients without overhydration, while the CAPD patients with CVD were more fluid overloaded than the non CVD patients. Moreover, the overhydrated CAPD patients had a higher cardiac event rate and tended to have a poor patient survival than that of the normal hydrated CAPD patents in this study. The relationship among overhydration, systolic blood pressure, and CVD as well as the role of all these three factors in the pathogenesis of mortality need further investigation in PD patients. On the other hand, a substantial proportion of patients did not follow this paradigm. Some PD patients had a normal blood pressure despite being fluid overloaded. These patients either suffer from congestive heart failure, or had a lower total peripheral resistance [2], [3], [40]. Some PD patients had uncontrolled hypertension despite normal tissue hydration. It is postulated that they are probably patients suffered from vascular stiffness or having a higher total peripheral resistance [2], [3]. Of note, thirty-two (10.4%) patients in our study were over hydrated and normotensive but taking more than 2 anti-hypertensive drugs. Compared with the other normotensive patients, these 32 patients had a lower residual urine output (data no shown, *P* = 0.044), and used higher glucose concentration solution (*P* = 0.011), which suggested us to strengthen fluid removal in these patients to achieve the aim of maintaining normal blood pressure without useage of antihypertensive agents [41].

Although Konings CJ et al demonstrated that fluid status was related to peritoneal transport characteristics [42], we failed to demonstrate this relationship in our study. This result is consistent with Van Biese et al [2], who found a weak link between them, and disappearing in the multivariate analysis. Nevertheless, peritoneal transport status can help us understanding the mechanism of ultrafiltration failure and choosing appropriated management. After recognizing the high prevalence of fluid overload and related factors in the CAPD patients, we suggest that improving patient’s malnutrition, protecting residual renal function and increasing residual urine output may improve patient’s fluid status. Actually, all the associations between fluid overload and modifiable risk factors shown in our study may enhance clinical opportunities to improve morbidity and mortality in CAPD patients by identifying early overhydration and adopting measures to improve fluid status. Moreover, our survey supported that the BIA measurement could be used as a simple bedside screening tool in clinical evaluation of fluid overload in CAPD patients.

### Limitation

The limitations of this report should also be noted. This is a single center cross-sectional study, and the definition of fluid overload comes from the BIA device manufacturer but lack of Chinese healthy control data. Also, patients’ sodium intake restriction was only educated by the staff but not calculated accurately in this study. Further studies are needed to confirm the causal factors of fluid overload and impact of fluid control guided by BIA on residual renal function, nutrition, and cardiovascular function in CAPD patients.

### Conclusion

In conclusion, fluid overload is common in CAPD patients. Bioimpedance analysis monitoring can help to identify early and occult fluid overload in CAPD patients. Fluid overload is independently associated with the protein-energy wasting, old age, higher systolic blood pressure, reduced residual urine volume, and hypokalemia in CAPD patients. CAPD patients with fluid overload have a higher cardiac event rate after 1 year and tend to have poor 1- patient survival than that of the normal hydrated CAPD patents. Further study is needed to evaluate whether monitoring fluid balance can improve residual renal function, nutrition and patient outcome.
